# 5,6-Di­hydro-1,4-dithiine-2,3-di­carb­oxy­lic anhydride

**DOI:** 10.1107/S2414314623006478

**Published:** 2023-08-04

**Authors:** Olivia Bullock, Sarah Rice, Marcus R. Bond

**Affiliations:** a Department of Chemistry and Physics, Southeast Missouri State University, Cape Girardeau, MO 63701, USA; University of Aberdeen, United Kingdom

**Keywords:** crystal structure, fused ring, heterocycle, dithiine, anhydride

## Abstract

In the title com­pound (systematic name: 2,3-di­hydro-1,4-dithiino[2,3-*c*]furan-5,7-dione), C_6_H_4_O_3_S_2_, the observed geometry agrees well with those of its phthalamide, thieno and hy­droxy analogs, and with a calculated geometry obtained by density functional theory (DFT) calculations. Specific structural features are an S—C—C—S torsion angle of −70.39 (17)° and S—C bonds to *sp* ^2^-hybridized C atoms approximately 0.1 Å shorter than those to *sp* ^3^-hybridized C atoms. Unlike the extended structures of the analogs, there are no directed inter­molecular inter­actions and the head-to-tail rows of mol­ecules that are a prominent structural motif of the packing can be rationalized in terms of optimized dipole–dipole inter­actions.

## Abstract 2

The heterocyclic fused ring geometry of the title com­pound coincides with the geometries of related mol­ecules but without directed inter­molecular contacts determining the crystal packing.

## Graphical abstract

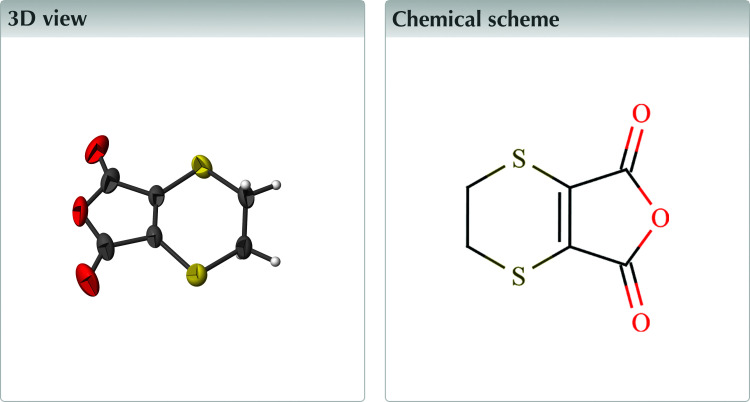

## Structure description

The unit-cell parameters for the title com­pound have been reported previously [Grabowski, 1968 ▸; Cambridge Structural Database (CSD; Groom *et al.*, 2016 ▸) refcode QQQDIA], but atomic coordinates are not available. Related com­pounds with reported three-dimensional atomic coordinates are the phthalamide (DTHPIM; Kirfel *et al.*, 1975 ▸), the thieno (ZUHQUQ; Skabara *et al.*, 2003 ▸) and the monohy­droxy (USUMOL; Kurbangalieva *et al.*, 2010 ▸) analogs, all of which are reported to crystallize in the monoclinic space groups *P*2_1_/*c* or *P*2_1_/*n*. We report here the three-dimensional structure of 5,6-di­hydro-1,4-dithiine-2,3-di­carb­oxy­lic anhydride, which crystallizes in the triclinic space group *P* 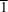 with unit-cell parameters in agreement with those reported by Grabowski.

**Figure 1:**
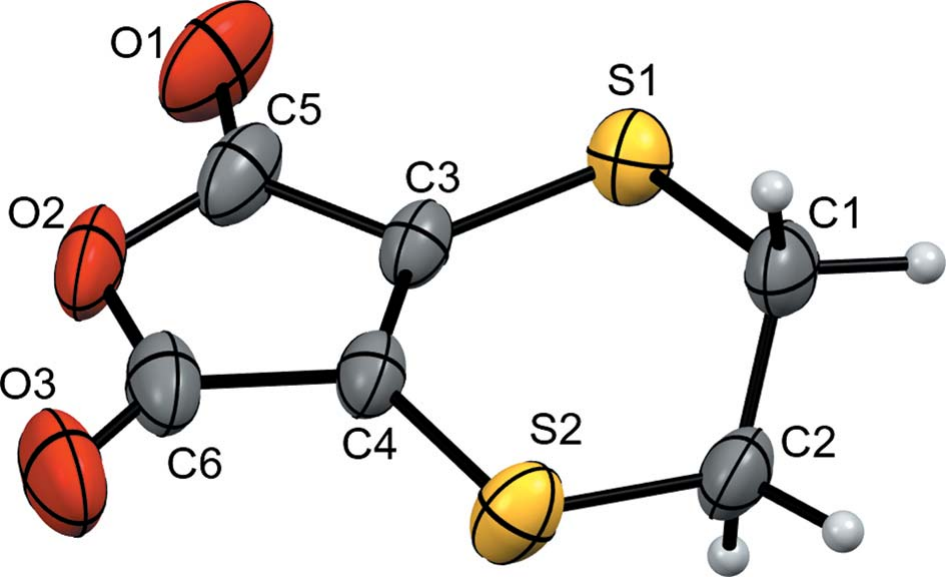
Displacement ellipsoid plot at the 50% probability level of the formula unit of the title com­pound, showing labels for non-H atoms.

The mol­ecule (Fig. 1 ▸) consists of furan­dione and di­hydro-1,4-dithiine rings fused by a common carbon–carbon double bond (atoms C3 and C4) and is largely planar (r.m.s. deviation of 0.044 Å from the mean plane for all atoms except the CH_2_ groups). The CH_2_ groups are twisted about the mol­ecular plane in order to reduce angle strain, with C1 0.323 (3) Å above and C2 0.528 (3) Å below, and an S1—C1—C2—S2 torsion angle of −70.39 (17)°. The S—C bond lengths are 0.096 (7) Å shorter for bonds to *sp* ^2^-hybridized C atoms than to those with *sp* ^3^-hybridization (Table 1 ▸). The inter­ior bond angles within the furan­dione ring are close to idealized values for a uniform penta­gon, ranging from 107.44 (17) to 108.36 (18)°. The O=C—O angles have expected values of 121–122° for an *sp* ^2^-hybridized center, while the external O=C—C angles average 130.2 (7)° in order to accommodate the geometry of the planar ring. These details agree well with the geometrical parameters for maleic anhydride [MLEICA (Marsh *et al.*, 1962 ▸) and MLEICA01 (Lutz, 2001 ▸)].

**Figure 2:**
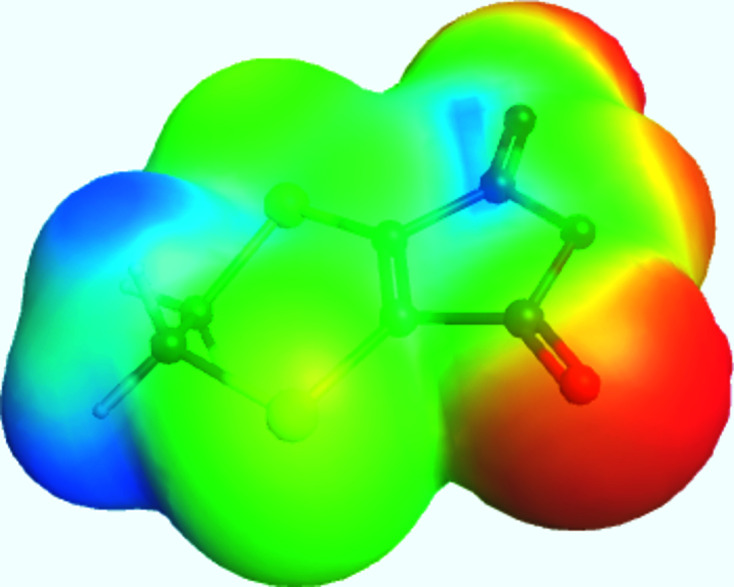
Electrostatic potential plot of the title mol­ecule with the optimized geometry visible. Red represents the most negatively charged regions, while blue represents the most positively charged.

The geometrical details for the related com­pounds listed above agree closely with those of the title com­pound. In particular, the S—C—C—S torsion-angle magnitudes range from 68.10 to 70.75° and the S—C bond lengths to *sp* ^2^-hybridized C atoms average 0.087 (14) Å shorter than those to *sp* ^3^-hybridized C atoms, with the phthalamide analog providing the closest agreement [average *sp* ^3^–*sp* ^2^ bond length difference = 0.0995 (7) Å]. A DFT geometry optimization *in vacuo* [B3LYP functional, cc-pTVZ basis set; *GAMESS* (Schmidt *et al.*, 1993 ▸)] yields similar results, with an S—C—C—S torsion angle of −69.6° and S—C bond lengths of 1.730 and 1.829 Å to *sp* ^2^- and *sp* ^3^-hybridized C atoms, respectively. An electrostatic potential plot with the optimized mol­ecule visible is presented in Fig. 2 ▸.

**Figure 3:**
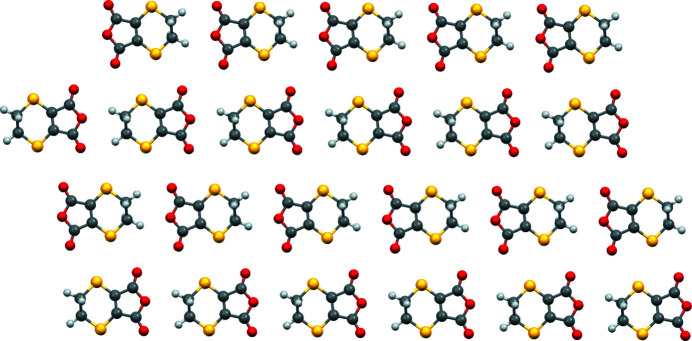
Ball-and stick diagram of the sheet structure viewed perpendiciular to (11 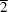 ).

**Figure 4:**
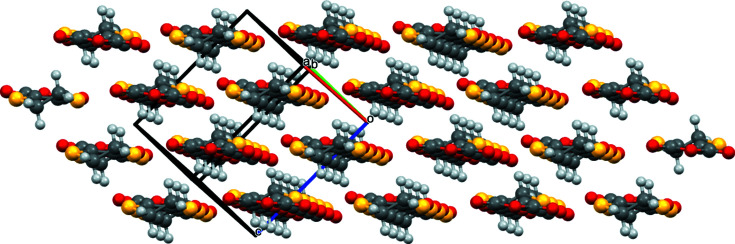
Ball-and-stick packing diagram of a unit cell, with axis labels viewed along [1 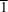 0], showing the stacking of four sheets to generate the three-dimensional structure.

The unit-cell packing of the title com­pound consists of sheets of mol­ecules lying parallel to (11 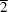 ), with neighboring sheets related by inversion. The mol­ecular planes are approximately coplanar with the sheet, with mol­ecules forming head-to-tail rows parallel to [1 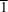 0] within the sheet. Neighboring rows within the sheet have opposite orientations, while rows on neighboring sheets straddle each other. This packing differs from that of similar mol­ecules, where directed hydrogen bonding or short S⋯O contacts feature prominently, with the head-to-tail rows of mol­ecules in the title com­pound rationalized in terms of optimized dipole–dipole inter­actions. A ball-and-stick diagram of a sheet is presented in Fig. 3 ▸ and a unit-cell packing diagram viewed edge on to the sheets is presented in Fig. 4 ▸.

## Synthesis and crystallization

5,6-Di­hydro-1,4-dithiin-2,3-di­carb­oxy­lic anhydride (98+%) was purchased from Fisher Scientific and recrystalized by slow evaporation at room temperature from tetra­hydro­furan solution to yield yellow block-like crystals.

## Refinement

Crystal data, data collection, and structure refinement details are listed in Table 2 ▸. The final structure refinement was carried out within the *OLEX2* system *via* Hirshfeld atom refinement with nonspherical atomic form factors using *NoSpherA2* (Kleemiss *et al.*, 2021 ▸; Midgley *et al.*, 2021 ▸) derived from electron density from DFT calculations using *ORCA* (B3LYP functional, def2-SVP basis set; Neese, 2022 ▸). All atoms were refined anisotropically.

## Supplementary Material

Crystal structure: contains datablock(s) I, global. DOI: 10.1107/S2414314623006478/hb4438sup1.cif

Structure factors: contains datablock(s) I. DOI: 10.1107/S2414314623006478/hb4438Isup2.hkl

Supporting information file. DOI: 10.1107/S2414314623006478/hb4438Isup3.cml

CCDC reference: 2284875

Additional supporting information:  crystallographic information; 3D view; checkCIF report

## Figures and Tables

**Table 1:** Selected geometric parameters (Å, °)

S1—C1	1.805 (2)	C3—C5	1.469 (3)
S1—C3	1.7136 (19)	C4—C6	1.472 (3)
S2—C2	1.817 (2)	O1—C5	1.193 (3)
S2—C4	1.7160 (19)	O2—C5	1.376 (3)
C1—C2	1.510 (3)	O2—C6	1.386 (3)
C3—C4	1.345 (3)	O3—C6	1.186 (3)
			
C1—S1—C3	99.54 (10)	C3—C4—C6	107.44 (17)
C2—S2—C4	98.30 (10)	O1—C5—C3	129.7 (2)
S1—C1—C2	114.41 (15)	O2—C5—C3	108.36 (18)
C1—C2—S2	115.01 (15)	O1—C5—O2	121.98 (19)
S1—C3—C4	131.57 (14)	O2—C6—C4	108.13 (18)
S1—C3—C5	120.60 (16)	O3—C6—C4	130.7 (2)
C4—C3—C5	107.83 (17)	O2—C6—O3	121.2 (2)
C3—C4—S2	129.27 (14)	C5—O2—C6	108.14 (15)
C6—C4—S2	123.28 (16)		

**Table 2:** Experimental details

Crystal data	
Chemical formula	C_6_H_4_O_3_S_2_
*M*_r_	188.23
Crystal system, space group	Triclinic, *P* 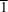
Temperature (K)	295
*a*, *b*, *c* (Å)	5.398 (1), 7.5537 (15), 9.2566 (18)
α, β, γ (°)	89.273 (6), 87.361 (6), 75.701 (5)
*V* (Å^3^)	365.36 (12)
*Z*	2
Radiation type	Mo *K*α
μ (mm^−1^)	0.68
Crystal size (mm)	0.42 × 0.38 × 0.17

Data collection	
Diffractometer	Bruker D8 Quest Eco
Absorption correction	Multi-scan (*SADABS*; Bruker, 2016 ▸)
*T*_min_, *T*_max_	0.648, 0.746
No. of measured, independent and observed [*I* ≥ 2u(*I*)] reflections	16978, 1669, 1338
*R*_int_	0.046
(sin θ/λ)_max_ (Å^−1^)	0.651

Refinement	
*R*[*F* ^2^ > 2σ(*F* ^2^)], *wR*(*F* ^2^), *S*	0.033, 0.074, 1.11
No. of reflections	1669
No. of parameters	136
H-atom treatment	All H-atom parameters refined
Δρ_max_, Δρ_min_ (e Å^−3^)	0.45, −0.25

Computer programs: *APEX3* and *SAINT* (Bruker, 2018 ▸), *SHELXT2014* (Sheldrick, 2015*a* ▸), *SHELXL2016* (Sheldrick, 2015*b* ▸), *olex2.refine* (Bourhis *et al.*, 2015 ▸), *OLEX2* (Dolomanov *et al.*, 2009 ▸), *Mercury* (Macrae *et al.*, 2020 ▸), *WebMO* (Schmidt & Polik, 2016 ▸), and *publCIF* (Westrip, 2010 ▸).

## full crystallographic data

### Crystal data

**Table:**

C_6_H_4_O_3_S_2_	*Z* = 2
*M**_r_* = 188.23	*F*(000) = 192.603
Triclinic, *P*1	*D*_x_ = 1.711 Mg m^−^^3^
*a* = 5.398 (1) Å	Mo *K*α radiation, λ = 0.71073 Å
*b* = 7.5537 (15) Å	Cell parameters from 7641 reflections
*c* = 9.2566 (18) Å	θ = 2.8–27.4°
α = 89.273 (6)°	µ = 0.68 mm^−^^1^
β = 87.361 (6)°	*T* = 295 K
γ = 75.701 (5)°	Plate, yellow
*V* = 365.36 (12) Å^3^	0.42 × 0.38 × 0.17 mm

### Data collection

**Table:**

Bruker D8 Quest Eco diffractometer	1338 reflections with *I*≥ 2u(*I*)
φ and ω scans	*R*_int_ = 0.046
Absorption correction: multi-scan (SADABS; Bruker, 2016)	θ_max_ = 27.6°, θ_min_ = 3.6°
*T*_min_ = 0.648, *T*_max_ = 0.746	*h* = −7→7
16978 measured reflections	*k* = −9→9
1669 independent reflections	*l* = −12→12

### Refinement

**Table:**

Refinement on *F*^2^	0 constraints
Least-squares matrix: full	Primary atom site location: dual
*R*[*F*^2^ > 2σ(*F*^2^)] = 0.033	All H-atom parameters refined
*wR*(*F*^2^) = 0.074	*w* = 1/[σ^2^(*F*_o_^2^) + (0.0229*P*)^2^ + 0.1706*P*] where *P* = (*F*_o_^2^ + 2*F*_c_^2^)/3
*S* = 1.11	(Δ/σ)_max_ = 0.0002
1669 reflections	Δρ_max_ = 0.45 e Å^−^^3^
136 parameters	Δρ_min_ = −0.25 e Å^−^^3^
0 restraints	

### Fractional atomic coordinates and isotropic or equivalent isotropic displacement parameters (Å^2^)

**Table:**

	*x*	*y*	*z*	*U*_iso_*/*U*_eq_	
S1	0.87227 (9)	0.35661 (7)	0.38382 (6)	0.04127 (16)	
S2	0.45568 (10)	0.23942 (8)	0.13318 (6)	0.04824 (17)	
C1	0.8444 (5)	0.1333 (3)	0.3315 (2)	0.0440 (5)	
H1a	1.028 (5)	0.039 (4)	0.347 (3)	0.091 (10)	
H1b	0.700 (5)	0.098 (3)	0.400 (3)	0.072 (8)	
C2	0.7794 (4)	0.1195 (3)	0.1758 (2)	0.0440 (5)	
H2a	0.793 (6)	−0.019 (4)	0.152 (3)	0.086 (9)	
H2b	0.909 (5)	0.169 (4)	0.102 (3)	0.064 (8)	
C3	0.6129 (3)	0.4890 (2)	0.3010 (2)	0.0338 (4)	
C4	0.4505 (3)	0.4463 (2)	0.2093 (2)	0.0347 (4)	
C5	0.5328 (4)	0.6869 (3)	0.3268 (2)	0.0457 (5)	
C6	0.2545 (4)	0.6150 (3)	0.1802 (2)	0.0478 (5)	
O1	0.6268 (4)	0.7808 (2)	0.3987 (2)	0.0682 (5)	
O2	0.3162 (3)	0.75751 (18)	0.25156 (17)	0.0558 (4)	
O3	0.0695 (3)	0.6399 (2)	0.11090 (19)	0.0695 (5)	

### Atomic displacement parameters (Å^2^)

**Table:**

	*U*^11^	*U*^22^	*U*^33^	*U*^12^	*U*^13^	*U*^23^
S1	0.0364 (3)	0.0389 (3)	0.0489 (3)	−0.0100 (2)	−0.0021 (2)	−0.0005 (2)
S2	0.0401 (3)	0.0441 (3)	0.0610 (4)	−0.0107 (2)	−0.0014 (2)	−0.0144 (3)
C1	0.0523 (14)	0.0263 (10)	0.0484 (13)	−0.0007 (10)	0.0016 (11)	0.0030 (9)
H1a	0.08 (2)	0.057 (19)	0.10 (2)	0.045 (17)	−0.031 (18)	0.019 (17)
H1b	0.09 (2)	0.033 (15)	0.10 (2)	−0.030 (15)	0.015 (18)	−0.009 (15)
C2	0.0464 (12)	0.0299 (11)	0.0496 (13)	0.0014 (9)	0.0061 (10)	−0.0076 (10)
H2a	0.13 (3)	0.038 (16)	0.09 (2)	−0.012 (17)	0.007 (18)	−0.021 (15)
H2b	0.053 (17)	0.076 (19)	0.060 (17)	−0.013 (15)	0.022 (13)	0.014 (15)
C3	0.0335 (9)	0.0236 (9)	0.0427 (10)	−0.0055 (7)	0.0074 (8)	−0.0008 (8)
C4	0.0306 (9)	0.0281 (9)	0.0415 (10)	−0.0011 (7)	0.0060 (8)	0.0003 (8)
C5	0.0551 (13)	0.0245 (10)	0.0556 (13)	−0.0096 (9)	0.0171 (10)	−0.0021 (9)
C6	0.0370 (11)	0.0442 (12)	0.0528 (12)	0.0054 (9)	0.0076 (10)	0.0118 (10)
O1	0.0867 (13)	0.0358 (9)	0.0862 (12)	−0.0262 (9)	0.0178 (10)	−0.0185 (9)
O2	0.0611 (10)	0.0270 (7)	0.0673 (10)	0.0084 (7)	0.0161 (8)	0.0070 (7)
O3	0.0467 (9)	0.0712 (12)	0.0784 (12)	0.0086 (8)	−0.0076 (9)	0.0218 (10)

### Geometric parameters (Å, º)

**Table:**

S1—C1	1.805 (2)	C2—H2b	1.08 (2)
S1—C3	1.7136 (19)	C3—C4	1.345 (3)
S2—C2	1.817 (2)	C3—C5	1.469 (3)
S2—C4	1.7160 (19)	C4—C6	1.472 (3)
C1—H1a	1.08 (2)	O1—C5	1.193 (3)
C1—H1b	1.06 (2)	O2—C5	1.376 (3)
C1—C2	1.510 (3)	O2—C6	1.386 (3)
C2—H2a	1.06 (2)	O3—C6	1.186 (3)
			
C1—S1—C3	99.54 (10)	S1—C3—C4	131.57 (14)
C2—S2—C4	98.30 (10)	S1—C3—C5	120.60 (16)
S1—C1—H1a	107.2 (15)	C4—C3—C5	107.83 (17)
S1—C1—H1b	108.1 (12)	C3—C4—S2	129.27 (14)
H1a—C1—H1b	110 (2)	C6—C4—S2	123.28 (16)
S1—C1—C2	114.41 (15)	C3—C4—C6	107.44 (17)
C2—C1—H1a	107.8 (16)	O1—C5—C3	129.7 (2)
C2—C1—H1b	108.9 (15)	O2—C5—C3	108.36 (18)
C1—C2—S2	115.01 (15)	O1—C5—O2	121.98 (19)
S2—C2—H2a	105.1 (17)	O2—C6—C4	108.13 (18)
S2—C2—H2b	107.5 (13)	O3—C6—C4	130.7 (2)
C1—C2—H2a	108.8 (16)	O2—C6—O3	121.2 (2)
C1—C2—H2b	111.8 (14)	C5—O2—C6	108.14 (15)
H2a—C2—H2b	108 (2)		
			
S1—C1—C2—S2	−70.39 (17)	S2—C4—C6—O3	4.1 (2)
S1—C3—C4—S2	−3.9 (2)	C3—C4—C6—O2	3.05 (16)
S1—C3—C4—C6	176.69 (19)	C3—C4—C6—O3	−176.50 (17)
S1—C3—C5—O1	1.95 (19)	C3—C5—O2—C6	0.24 (18)
S1—C3—C5—O2	−177.86 (15)	C4—C6—O2—C5	−1.95 (18)
S2—C4—C3—C5	176.57 (18)	C5—O2—C6—O3	177.65 (17)
S2—C4—C6—O2	−176.39 (17)		
